# Intimate partner violence and exclusive breastfeeding of infants: analysis of the 2013 Nigeria demographic and health survey

**DOI:** 10.1186/s13006-021-00361-9

**Published:** 2021-01-23

**Authors:** Tolulope Ariyo, Quanbao Jiang

**Affiliations:** Institute for Population and Development Studies, School of Public Policy and Administration, Xi’an Jiaotong University, Shaanxi, China

**Keywords:** Breastfeeding, Infant feeding, Domestic violence, Physical violence, Psychological violence, Sexual violence, Nigeria

## Abstract

**Background:**

Existing knowledge on the relationship between intimate partner violence (IPV) and exclusive breastfeeding (EBF) in the context of Nigeria is minimal and limited to a lifelong measure of IPV experience. An abuse experienced a long time ago may not have as much negative effect as that encountered at a more proximal time to the breastfeeding phase. To this effect, we examined this relationship with maternal IPV experienced around the time of pregnancy and postpartum.

**Method:**

We analyzed data from the 2013 Nigeria Demographic and Health Survey. The sample includes 2668 breastfeeding mothers having a child aged under 6 months. The outcome variable was EBF or mixed-feeding (24 h recall). The exposure variables were: the maternal experience of psychological, physical, and sexual intimate partner violence. Also, there was an experience of any form of IPV and frequency score of intimate partner violence. Analysis includes chi-square and t-test bivariates, complete case and imputed logistic regressions for binary outcome.

**Results:**

In the imputed analysis, compared to mothers who experienced no IPV, those who experienced IPV had a 26% reduced likelihood of EBF practice (AOR 0.74; 95% CI 0.55, 1.00). Also, a unit dose of maternal IPV experience was associated with a 5% reduced likelihood of EBF practice (AOR 0.69; 95% CI 0.49, 0.98). Among the three forms of IPV, physical IPV had the highest effect size. Physical IPV was associated with a 37% reduced likelihood of EBF practice (AOR 0.63; 95% CI 0.44, 0.90), while psychological IPV was associated with a 34% reduced likelihood of EBF practice (AOR 0.66; 95% CI 0.47, 0.92), when compared to the respective reference groups. On the other hand, those who reported sexual IPV were just as likely to breastfeed as those who did not (AOR 0.94; 95% CI 0.62, 1.41).

**Conclusions:**

In this study, maternal IPV is associated with EBF practice. Policies aimed at promoting EBF should also be framed to combat IPV against pregnant women and nursing mothers.

## Background

While the initiation of breastfeeding in the first hour of birth is around 50% across most developing countries [1], the rate of exclusive breastfeeding (EBF) practice is much lower, and just 23% in Nigeria [2]. Exclusive breastfeeding refers to feeding a young infant only breastmilk for the first 6 months of life [3, 4]. The medical benefit of this practice especially for infants, has been reported to include strengthening of the immune system and reduction of the risk of morbidity [5–7]. However, the decision of whether to continue breastfeeding exclusively hinges on various social, psychological, emotional, and environmental factors [8]. Women who cohabitate in an abusive relationship as victims of intimate partner violence (IPV) have been known to develop depressive symptoms or other severe health issues [9–12]. Intimate partner violence refers to the abuse or aggression between people involved in an intimate relationship [13]. About one in three of ever-married women in Nigeria are reported to have experienced physical, sexual, or emotional intimate partner violence [14].

The perpetration of IPV could go in either or in both directions, but when nursing mothers are the victims rather than perpetrators, the consequent mental or emotional distress could impair adequate childcare duties [15, 16]. Evidence has also suggested that an infant’s exposure to IPV could pose a risk of trauma or psychopathology in early infancy. Studies conducted to examine the multiple forms of traumata in infants, including IPV, found that witnessing a threat to a caregiver was related to severe symptoms of increased hyperarousal and fear [17, 18].

Research from the developed countries that have examined the relationship between IPV and EBF have reported mixed findings. For instance, while studies from Spain and the United States of America have found an association between IPV and EBF [19–21], studies from Australia and Sweden have reported that there is no association between the two [22, 23]. This dissimilarity could be a result of the differences in the type of samples used. While some were based on a sample from a national survey, others were based on a sample involving participants in a program at a health institution. Studies from the developing countries on the other hand, specifically from Southern Asia, have mostly reported associations [24, 25]. In the context of sub-Sahara Africa, only one study had examined this relationship. In that study, which was a comparative analysis involving eight African countries, only the result for Nigeria showed no adjusted association between EBF and all the forms of IPV which was measured from lifelong experience [26].

In respect to the timing of the event, IPV could be of multiple variants such as lifelong experience, pregnancy experience, or postpartum experience. Abuse experienced a long time ago may not have as much negative effect as that encountered at a more proximal time to the breastfeeding phase. Also, to what extent such experience is observed to affect mothering duties may depend on the characteristics of the study population [20]. The female literacy rate in Nigeria, which is one of the indicators of women empowerment shows a huge disproportion against women [27]. Therefore, to the effect that knowledge on the relationship between IPV and EBF in the context of Nigeria is not yet fully established, this study aims to re-examine this relationship, but with a focus on IPV measured from pregnancy and postpartum experiences.

## Methods

### Data source and study design

This study analyzed data collected from the 2013 Nigeria Demographic and Health Survey (DHS). The information was collected from February to July 2013. Nigeria is Africa’s most populous country with an estimated population of over 200 million people of diverse ethnic and cultural backgrounds [28]. The National Population Commission (NPC) in collaboration with ICF Macro, Calverton, MD, USA conducted the survey. The sampling involved a three-stage cluster design. This consisted of a selection of 904 primary sampling units (PSUs), 372 in the urban, and 532 in the rural. A nationally representative sample of 40,680 households was then selected across the PSUs. Both married men and women in the households were eligible to be interviewed with the corresponding version of the questionnaire designed for males and females separately. About 39,902 women aged 15 to 49 years were identified as eligible of which 98% were successfully interviewed with the women’s version of the questionnaire. Questions were asked relating to household sociodemography, maternal health, as well as child wellbeing. The IPV module was a subsample survey within this general survey and it was based on a shortened and modified version of the Conflict Tactics Scale (CTS) [29]. This modification to the original scale was done between 1998 and 99 by ICF Macro, the organizer of the DHS programs, after consultation with experts on domestic violence measurement, gender, and survey research [30]. It was subsequently tested and validated through pilot studies in Cambodia, Colombia, and Haiti in 2000, and then The Dominican Republic in 2002 [30]. Concurrent validity has also been established for this modified scale, as it has been used for the DHS programs implemented in over 90 countries afterward across Africa, Asia, and Latin America. Additionally, studies utilizing data from those surveys have consistently reported a high Cronbach alpha indicating an internal reliability of construct [31–34]. The advantage of the DHS program’s modified CTS includes the fact that it incorporates questions on sexual violence alongside physical violence, and also does not assume that violence takes place only in situations characterized by conflict.

During the survey, only one woman per household was selected for the IPV module. Specially constructed weights were used to adjust for this selection pattern to ensure that the IPV subsample was nationally representative. Three specific protections were built into the survey questionnaire under the World Health Organization’s (WHO) ethical and safety recommendations [35]. These include the informed consent of the respondent, privacy during the inquiry, and confidentiality of the information shared. The team of interviewers comprised four females and two males who had been equipped with the necessary training to conduct the IPV module. The questionnaire was originally designed in English, but before its implementation for the survey in Nigeria, it was also translated into the three major Nigerian languages—Hausa, Igbo, and Yoruba, by the NPC, through a stakeholder meeting in March 2012. It was pretested, refined, and finalized for the survey. More information about the survey setting, and data collection is provided in a final report [14].

For our study, sample selection was limited to women who were interviewed in the IPV module, currently resides with her partner, had a child under 6 months of age who also resides with her, and the woman indicated to be currently breastfeeding. A total of 2668 mother-infant dyads met these criteria.

### Variables and measures

The outcome variable of interest was binary, indicating if a breastfeeding infant under 6 months of age was undergoing EBF (=1) or mixed-feeding (=0). This was determined by questions on whether the child was given certain types of solid or semi-solid food in the prior 24 h to the survey. These were typically foods that were not recommended for infants under 6 months. Only those who responded not to have fed the child with any of the listed food items other than breast milk were regarded to be practicing exclusive breastfeeding.

The exposure variables were forms of IPV measured through 13 questions contained in the DHS program’s modified CTS known as the DHS domestic violence module [30]. It bordered on possible violent events a woman experienced from a current male partner in the last 12 months (Table 1). It was assumed that the time period captured events that may have occurred during pregnancy or postpartum.

**Table 1:** The list of question items and response pattern on IPV

		Response	
S/N	Items	No	Yes
1	Ever been humiliated by husband/partner	0	1
2	Ever been threatened with harm by husband/partner	0	1
3	Ever been insulted or made to feel bad by husband/partner	0	1
4	Ever been pushed, shaken or had something thrown by husband/partner	0	1
5	Ever been slapped by husband/partner	0	1
6	Ever been punched with fist or hit with something harmful by husband/partner	0	1
7	Ever been kicked or dragged by husband/partner	0	1
8	Ever been strangled or burnt by husband/partner	0	1
9	Ever been threatened with knife/gun or other weapon by husband/partner	0	1
10	Ever had arm twisted or hair pulled by husband/partner	0	1
11	Ever been physically forced into unwanted sex by husband/partner	0	1
12	Ever been forced into other unwanted sexual acts by husband/partner (threats)	0	1
13	Ever been physically forced to perform sexual acts respondent did not want to	0	1

These questions were grouped into three forms of IPV. Psychological IPV involved three questions (items 1–3) with a Cronbach’s alpha of 0.73. Physical IPV involved seven questions (items 4–10) with a Cronbach’s alpha of 0.86. While sexual IPV involved three questions (items 11–13) with a Cronbach’s alpha of 0.86. A combined variable was also created that included the experience of any of the forms of intimate partner violence. All four variables were binary coded. Additionally, the frequency of IPV was generated from the 13 possible events. This was used to measure the dose-effect of an IPV experience. The questions in the survey had asked about the frequency of occurrence of each violence. The responses include; Never (=0), Sometimes (=1), and Often (=2). This, therefore, yielded a scale from 0 (no violent events in the past year) to 26 (experiencing every violent event often in the past year).

Some of the covariates adopted in this study have been discussed in the literature as possible determinants of exclusive breastfeeding [26, 36–38]. These include; child’s age measured in months; mother’s age measured in years; mother’s education measured as the number of years of formal education acquired; parity measured as the count of children a woman has; husband’s education measured as the number of years of formal education acquired by the partner; the number of other children under 5 years old in the household measured in count form; family wealth index constructed using household asset data via a principal component analysis. The family wealth index variable was already computed and available as part of the DHS dataset. Other covariates include mother’s employment status within the previous 12 months to the survey and we categorized this as binary (employed vs. not employed); the number of times antenatal care was attended based on WHO’s recommendation stipulating a minimum of four times, we grouped this as binary (less than 4 times vs. 4 times or more); metropolitan status grouped as binary (rural vs. urban) residency; and child’s size at birth as perceived by the mother grouped into 3 categories (small, medium and large). Since the child’s actual birthweight was not adequately captured in the survey, the mother’s perception of this was used as a proxy.

### Statistical analysis

We used non-weighted (sampling weights) cross-tabulation to present the distribution of the independent variables and covariates by the groups of EBF practice (EBF vs. mixed-feeding). We conducted both chi-square and t-test bivariate analyses to examine the association between the dependent variable and the independent variables. Chi-square to test for the relationship between variables and t-test for difference between means. To avoid the issue of multicollinearity, we conducted a diagnostic check between the independent variables and covariates, all variance inflation factors (VIF) were below 10, with an average VIF of 1.82.

Furthermore, we performed two logistic regression analyses. One was a complete case analysis that only included observation with no missing values (*N* = 2465). The second regression was a multiple imputation analysis where the missing observation of 7.6% was imputed (*N* = 2668). This was to help determine if there had been any serious attenuations as a result of the case-wise deletion of observations and to also make use of all available information in the selected sample. We conducted this imputation using the Markov Chain Monte Carlo (MCMC) method [39, 40], under the assumption of missing at random (MAR) [40]. We specified 10 imputations, and this was deemed sufficient to re-create the variance-covariance estimation as indicated by the Monte Carlo error check [39, 41]. Each of the regression methods contained five models, each model had one independent variable with covariates. Stata version 15.1 was used to implement the data imputation, as well as to conduct all analyses. All regression analyses were done using the survey design (sampling weights). The adjusted odds ratio was reported at the 95% significance level threshold.

## Results

### Characteristics of respondents

As shown in Table 2, the mean age of respondents was 28 years (SD = 6.5), the average years of education attained was 5 years (SD = 5.5), and majority (62.4%) were not employed. In regards to their fertility, a greater proportion (83%) were multiparous, and a little more than half (55%) reported to have attended antenatal care four times or more during the pregnancy of their last child which was included in the dyad selection. The mean age of the children was about 2 months (SD = 1.1) and the majority of them (the children) 84.7% were perceived to be of an average or large size at birth. A greater proportion of the respondents (64.8%) were residents in rural areas.

**Table 2:** Non-weighted cross-tabulation of descriptive characteristics of respondents by EBF practice

	Overall		Exclusive Breastfeeding					
	***N***	%	No	%	Yes	%	Mean	SD
**Control Variables**								
Age							28	6.5
Education (In years)							5	5.5
Partner’s Education (In years)							7	6.8
Employment status								
Currently Employed	928	37.7	342	37.0	586	38.0		
Not Employed	1537	62.4	582	63.0	955	62.0		
Wealth Index							−0.20	1.0
Antenatal								
Less than 3	1120	45.4	403	43.6	717	46.5		
More than 3	1345	54.6	521	56.4	824	53.5		
Parity								
Primiparous	454	18.4	158	17.1	296	19.2		
Multiparity	2011	81.6	766	82.9	1245	80.8		
Child age							3	1.9
No. of U5							2.2	1.1
Perceived size								
Very Small	378	15.3	139	15.0	239	15.5		
Average	985	40.0	343	37.1	642	41.7		
Large	1102	44.7	442	47.8	660	42.8		
Residence								
Rural	867	64.8	327	64.6	540	65.0		
Urban	1598	35.2	597	35.4	1001	35.0		

Total observations =2465, Weights refer to sampling weights, *SD*= Standard deviation, *U5*= Other children under 5years old in the household

### Univariate and bivariate

The prevalence of IPV among the study sample was 21.1%, with psychological IPV being the most reported (16.6%) and sexual IPV the least (4.6%). Physical IPV on the other hand had a prevalence of 11.7%. The average frequency score of IPV among the respondents was 0.69 (SD = 2.0). Among the exposure variables, the chi-square bivariate only indicated a significant relationship between physical IPV and EBF at a 95% significance level (Table 3). Additionally, the t-test statistics also indicated a significant difference in the means of frequency score of IPV by the groups of EBF (EBF vs. mixed-feeding) at 95% significant level: *t*-test = 2.03; *p* < 0.05 (Table 3).

**Table 3:** Non- weighted cross-tabulation of maternal IPV by EBF practice

	Overall		Exclusive Breastfeeding							
	***N***	%	No	%	Yes	%	Chi-square	***P***-value	Mean	SD
**Independent Variables**										
Emotional IPV							2.48	0.12		
No	2057	83.5	757	81.9	1300	84.4				
Yes	408	16.6	167	18.1	241	15.6				
Physical IPV							4.11	**0.04**		
No	2176	88.3	800	86.6	1376	89.3				
Yes	289	11.7	124	13.4	165	10.7				
Sexual IPV							0.02	0.89		
No	2351	95.4	882	95.5	1469	95.3				
Yes	114	4.6	42	4.6	72	4.7				
Any IPV							0.06	0.80		
No	1946	79.0	727	78.7	1219	79.1				
Yes	519	21.1	197	21.3	322	20.9				
Frequency score of IPV							†2.45	**0.01**	0.69	2.0

Total observation = 2465, Weight refers to sampling weights, SD= Standard deviation

† t-test statistics

**Fig. 1:**
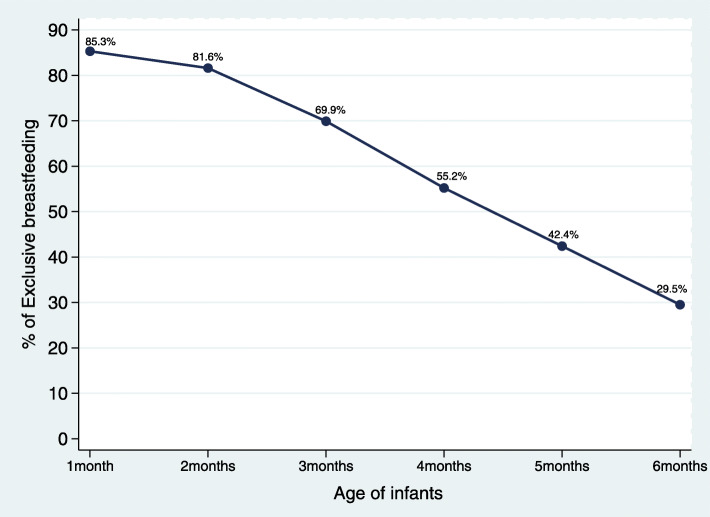
Rate of exclusive breastfeeding by age of infants

As shown in Fig. 1, the age of infants is negatively associated with the practice of exclusive breastfeeding. While over 85.3% of infants in the first month of life had EBF, it was only 55.2% among children in their third month of life and a further decline to 29.5% among children in their sixth month of life.

**Fig. 2:**
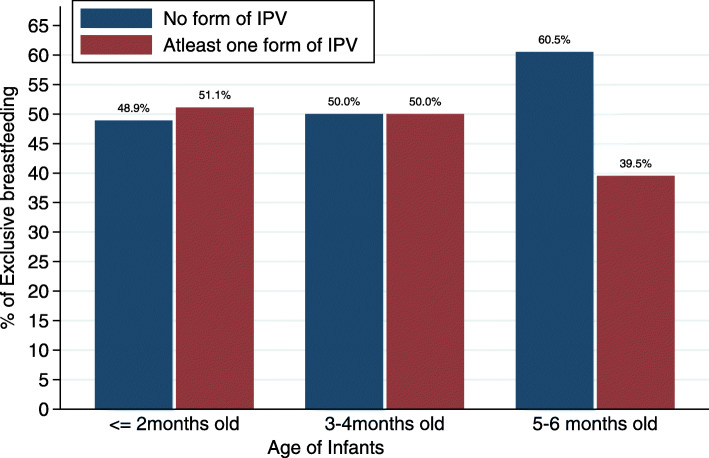
Rate of exclusive breastfeeding by maternal IPV and infant age

Furthermore, while the proportion of EBF practice among women who experienced any form of IPV does not clearly differ for children aged 2 months or less, and children 3–4 months old, the proportion of non-EBF practice was clearly higher among children 5–6 months old (Fig. 2).

### Result of regression analysis

The regression analyses examined the association between forms of IPV and EBF practice (EBF vs. mixed-feeding), while adjusting for covariates. In the complete case analysis, those who reported experiencing physical IPV had a 31% (AOR 0.73; 95% CI 0.53, 1.01; *p* < 0.05) reduced likelihood of EBF practice when compared against those who did not (Table 4, Model 2). Similarly, a unit increase in the frequency of IPV was associated with 5% (AOR 0.69; 95% CI 0.49, 0.98; *p* < 0.05) reduced likelihood of EBF practice (Table 4, Model 5). Although, the experience of psychological, sexual, and the combined variable (any form of IPV), showed the tendency of a reduced likelihood of EBF practice, but the effects were not statistically significant at the 95% threshold (Table 4, Models 1, 3, 4).

**Table 4:** Weighted logistic regression on the association between IPV and EBF (Complete cases)

Variables	Exclusive breastfeeding, AOR (CI)									
	Model 1		Model 2		Model 3		Model 4		Model 5	
Psychological IPV	0.73	(0.53, 1.01)	–	–	–	–	–	–	–	–
Physical IPV	–	–	0.69**	(0.49, 0.98)	–	–	–	–	–	–
Sexual IPV	–	–	–	–	0.94	(0.60, 1.46)		–	–	–
Any form of IPV	–	–	–	–	–	–	0.82	(0.61, 1.11)	–	–
IPV frequency score	–	–	–	–	–	–	–	–	0.95**	(0.90, 1.00)
Age	0.99	(0.97, 1.01)	0.99	(0.97, 1.01)	0.99	(0.97, 1.01)	0.99	(0.97, 1.01)	0.99	(0.97, 1.01)
Education	0.97**	(0.93, 1.00)	0.97*	(0.93, 1.00)	0.96**	(0.93, 1.00)	0.97**	(0.93, 1.00)	0.97**	(0.93, 1.00)
Age (Child)	0.62***	(0.59, 0.67)	0.63***	(0.59, 0.67)	0.63***	(0.59, 0.67)	0.63***	(0.59, 0.67)	0.63***	(0.59, 0.67)
Employed (Ref = No)	1.09	(0.87, 1.38)	1.09	(0.86, 1.37)	1.09	(0.86, 1.37)	1.09	(0.87, 1.38)	1.09	(0.87, 1.38)
Parity (Ref = Primipara)	0.84	(0.60, 1.17)	0.84	(0.60, 1.17)	0.83	(0.59, 1.16)	0.83	(0.60, 1.17)	0.83	(0.59, 1.16)
Antenatal (Ref = No)	0.91	(0.68, 1.21)	0.90	(0.68, 1.19)	0.89	(0.67, 1.18)	0.90	(0.68, 1.20)	0.90	(0.68, 1.19)
PBW (Ref = Big)										
Average	1.22	(0.93, 1.59)	1.22	(0.93, 1.59)	1.22	(0.93, 1.59)	1.22	(0.93, 1.59)	1.22	(0.93, 1.59)
Small	1.44**	(1.01, 2.06)	1.42*	(1.00, 2.03)	1.43**	(1.00, 2.04)	1.44**	(1.01, 2.05)	1.43**	(1.01, 2.05)
Education (Husband)	1.02	(0.99, 1.05)	1.02	(0.99, 1.05)	1.02	(0.99, 1.05)	1.02	(0.99, 1.05)	1.02	(0.99, 1.05)
Rural (Ref = Urban)	0.82	(0.57, 1.19)	0.81	(0.56, 1.18)	0.81	(0.56, 1.18)	0.82	(0.56, 1.18)	0.81	(0.56, 1.18)
Family wealth Index	1.02	(0.82, 1.26)	1.02	(0.82, 1.26)	1.03	(0.83, 1.27)	1.02	(0.82, 1.26)	1.02	(0.82, 1.26)
U5 children	1.05	(0.93, 1.19)	1.05	(0.93, 1.19)	1.05	(0.93, 1.19)	1.05	(0.93, 1.19)	1.05	(0.93, 1.19)
Constant	14.18***	(7.10, 28.34)	14.31***	(7.17, 28.59)	14.20***	(7.09, 28.45)	14.21***	(7.11, 28.40)	14.24***	(7.12, 28.48)
Observations	2465		2465		2465		2465		2465	

Weights refers to sampling weights

*** *p* < 0.01, ** *p* < 0.05

*AOR* Adjusted odds ratio

*CI* Confidence Interval

*Ref* Reference group

U5 Children No. of children under 5 years old in the household

*PBW* Perceived birthweight

The results of the imputed regression were similar in direction, but with a difference in magnitude. Additionally, statistical significance was retained for both psychological IPV and the combined variable (any form of IPV). Those who reported experiencing psychological IPV had 34% (AOR 0.66; 95% CI 0.47, 0.92; *p* < 0.05) reduced likelihood of EBF practice when compared against those who did not (Table 5, Model 1). While those who reported experiencing any form of IPV had 26% (AOR 0.74; 95% CI 0.55, 1.00; *p* < 0.05) reduced likelihood of EBF practice when compared against those who did not (Table 5, Model 4). Furthermore, the effect of physical IPV increased from 31 to 37% (AOR 0.63; 95% CI 0.44, 0.90; *p* < 0.05) (Table 5, Model 1), while the effect of a unit increase in the frequency of IPV remained unchanged. (Table 5, Model 5).

**Table 5:** Weighted logistic regression on the association between IPV and EBF (Multiple Imputation)

Variables	Exclusive breastfeeding, AOR (CI)									
	Model 1		Model 2		Model 3		Model 4		Model 5	
Psychological IPV	0.66**	(0.47, 0.92)	–	–	–	–	–	–	–	–
Physical IPV	–	–	0.63**	(0.44, 0.90)	–	–	–	–	–	–
Sexual IPV	–	–	–	–	0.94	(0.62, 1.41)	–	–	–	–
Any IPV	–	–	–	–	–	–	0.74**	(0.55, 1.00)	–	–
IPV frequency score	–	–	–	–	–	–	–	–	0.95**	(0.90, 1.00)
Age	0.99	(0.97, 1.01)	0.99	(0.97, 1.01)	0.99	(0.97, 1.01)	0.99	(0.97, 1.01)	0.99	(0.97, 1.01)
Education	0.96**	(0.93, 1.00)	0.96**	(0.93, 1.00)	0.96**	(0.93, 0.99)	0.96**	(0.93, 1.00)	0.96**	(0.93, 1.00)
Age (Child)	0.64***	(0.59, 0.68)	0.64***	(0.60, 0.68)	0.64***	(0.60, 0.69)	0.64***	(0.60, 0.68)	0.64***	(0.60, 0.68)
Employed (Ref = No)	1.06	(0.85, 1.33)	1.04	(0.83, 1.31)	1.05	(0.84, 1.32)	1.06	(0.84, 1.32)	1.06	(0.84, 1.32)
Parity (Ref = Primipara)	0.95	(0.72, 1.25)	0.93	(0.71, 1.22)	0.92	(0.70, 1.21)	0.94	(0.71, 1.23)	0.94	(0.71, 1.24)
Antenatal (Ref = No)	0.93	(0.67, 1.29)	0.93	(0.68, 1.29)	0.92	(0.67, 1.27)	0.93	(0.67, 1.28)	0.94	(0.68, 1.29)
PBW (Ref = Big)										
Average	1.23	(0.95, 1.60)	1.23	(0.95, 1.60)	1.23	(0.95, 1.60)	1.23	(0.95, 1.60)	1.24	(0.96, 1.60)
Small	1.33	(0.92, 1.93)	1.31	(0.90, 1.91)	1.32	(0.91, 1.92)	1.33	(0.91, 1.92)	1.33	(0.91, 1.93)
Education (Husband)	1.01	(0.98, 1.05)	1.01	(0.98, 1.05)	1.01	(0.98, 1.04)	1.01	(0.98, 1.04)	1.02	(0.99, 1.05)
Rural (Ref = Urban)	0.87	(0.61, 1.24)	0.85	(0.59, 1.22)	0.85	(0.59, 1.23)	0.86	(0.60, 1.23)	0.86	(0.60, 1.24)
Family wealth Index	1.00	(0.81, 1.24)	1.00	(0.81, 1.24)	1.02	(0.82, 1.26)	1.01	(0.82, 1.25)	1.01	(0.82, 1.24)
U5 children	1.03	(0.91, 1.16)	1.02	(0.91, 1.16)	1.02	(0.90, 1.15)	1.02	(0.90, 1.15)	1.03	(0.91, 1.16)
Constant	13.53***	(5.87, 31.18)	14.03***	(6.04, 32.59)	13.67***	(5.90, 31.71)	13.66***	(5.90, 31.63)	13.54***	(5.86, 31.28)
Observations	2668		2668		2668		2668		2668	

Weights refer to sampling weights

*** *p* < 0.01, ** *p* < 0.05

*AOR* Adjusted odds ratio

*CI* Confidence Interval

*Ref* Reference group

U5 Children No. of children under 5 years old in the household

*PBW* Perceived birthweight

## Discussion

With the use of the 2013 Nigeria DHS dataset, our study examined the association between IPV and the practice of EBF among nursing mothers in the context of Nigeria. In the results of our findings, the case-wise deletion of observation in the complete case analysis had slightly attenuated the effect of this relationship. The imputed analysis suggests that maternal IPV experienced around the time of pregnancy or postpartum is associated with suboptimal EBF practices. Except for sexual IPV, the two other forms of maternal IPV (psychological and physical IPV) were negatively associated with EBF practice, with physical IPV showing a higher magnitude. Furthermore, our findings also suggest that a dose experience of maternal IPV has a significant association with suboptimal breastfeeding. This indicates that multiple forms or repeated incidences of IPV during the time of pregnancy or postpartum is positively associated with suboptimal breastfeeding of young infants.

The current study contributes to knowledge by showing how different forms of IPV experienced around the time of pregnancy or postpartum is associated with exclusive breastfeeding of young infants in the context of Nigeria. To the best of our knowledge, this relationship had not been previously examined with a focus on IPV experienced around the time of pregnancy or postpartum period.

The findings of this study run somewhat contrary to that of Misch and Yount (2014) who, using the DHS data, reported that maternal IPV had no adjusted association with exclusive breastfeeding in Nigeria [26]. However, this difference is likely subject to two important factors. Firstly, while their study had used the 2008 Nigeria DHS data, we used a different dataset: the 2013 Nigeria DHS. Secondly, while their study had conceptualized IPV as a lifelong experience, we conceptualized it as that which is experienced around the time of pregnancy or postpartum. This goes to suggest that the proximity of the violence to the breastfeeding phase may be an important factor in determining an association. While events that happened a long time ago may or may not be associated with a mother’s ability or willingness to breastfeed her child, a violent event experienced during pregnancy of the child or postpartum period is likely to have an effect.

Furthermore, both psychological and physical IPV was associated with suboptimal breastfeeding. This finding which is a reflection of the deficient hypothesis [42, 43], was consistent with other cross-sectional studies from Bangladesh [6], USA [20], and India [24]. Mothers exposed to IPV may be less likely to breastfeed their infants optimally as a result of physiological or mental imbalance [44]. The path through which this happens could be in numerous forms. Firstly, women who are victims of IPV have been reported to be more at risk of depressive symptoms which could further lead to certain risk behaviors such as drinking, smoking, or drugs [45]. Substance abuse is associated with early discontinuation of breastfeeding either due to the potential danger for the child [46], or neglect in caregiving duties [47]. Secondly, according to the Nigeria DHS final report [14], 33% of ever-married women who had experienced spousal physical violence in the past 12 months, reported experiencing physical injuries. Even where the willingness is there to continue EBF, nursing mothers may not be able to do so if they had sustained serious injury from abuse. Thirdly, abusive husbands tend to be extremely possessive and controlling [48, 49]. Jealousy may sprout due to the volume of attention the mother gives the child. The mother may then be compelled to feed the child with infant formula due to lack of support from the partner who thinks that the breast is his property [50], or just out of concern that the child may not be getting enough milk.

In regards to sexual IPV, our findings suggest that nursing mothers who reported experiencing sexual violence are as likely to practice exclusive breastfeeding as those who reported not to have experienced sexual violence. However, while this result is consistent with the study of Metheny &, Stephenson (2019), who had also used a population-based study [24], it is different from that of Caleyachetty et al. (2019) who had used a pooled data of population-based studies across 51 low and middle-income countries (LMICs) [51]. The relatively small number of observations within this group in our study may have affected the result. It has also been established that physical violence in intimate relationships is more likely to be accompanied by psychological abuse rather than sexual abuse [45]. It could also be a case of differential reporting bias, owing to cultural reasons. While the survey questionnaire was carefully designed to capture lived experiences, and also pretested, capturing reports of rape and sexual violence still poses ethical and methodological challenges. One reason is the culture of silence regarding the incidence of rape due to the consequent stigmatization [14, 52, 53]. Another reason is about the patriarchal African culture characterized by male dominance and female subservience. This is believed to create some notion of male sexual entitlement [52], and as a result, women might be less likely to view unwanted sex as an act of violence.

The negative associative effect of maternal IPV experience on EBF suggests some policy implications for implementation. While a continual campaign against gender-based violence is obligatory, the victim’s confidence in the legal system to prosecute any reported case of violence is more likely to lead to reports of new violence [54]. Therefore, legal institutions should be adequately empowered to handle cases of violence against women. Furthermore, while it is necessary that screening for possible cases of IPV should be incorporated into antenatal and postnatal programs for pregnant women and nursing mothers respectively, it is also important to train nurses and midwives on identifying potential cases of abuse.

Although our study had not examined if maternal age moderates the relationship between IPV and EBF, but other studies using the Nigeria DHS data have established that women marrying at a young age is associated with the risk of IPV [55, 56]. Therefore, the practice of the girl-child marriage which is highly prevalent in certain parts of the country should be systematically discouraged. Union formation should not only be based on legal and physiological maturity, but more importantly, on mental maturity to deal with the uncertainties that may arise in a marriage union, as well as with the responsibilities of motherhood.

One of the major strengths of this study is the use of population-based data which gave room for generalization of findings. Additionally, the operationalization of IPV based on the experience of the past 12 months (prior to the survey) helped to keep within a proximal time frame thereby excluding events that might have happened over a long period and no longer having bearing on the current practices of breastfeeding. Also, our analysis examined the dose-effect of violence on the practice of exclusive breastfeeding.

However, the following limitations are associated with the survey and research design. Firstly, the use of cross-sectional data as with similar study designs, makes it difficult for any claims of causal relationships. Secondly, the variables used in our analysis were limited to what was captured by the survey. Specifically, variable on (postpartum) depressive symptoms was not captured. Had it been, its mediating role would have been examined. Thirdly, during the survey, violent experiences were captured as events that happened within the previous 12 months. There was no disassociation between events that happened during pregnancy or those that happen postpartum. As a result, the analysis was restricted from this dichotomy. Fourthly, due to the nature of the outcome variable of interest, bidirectional perpetration of violence was not considered in the operationalization of IPV. Being perpetrators may not be as likely to prevent a woman from breastfeeding as when they are victims. Lastly, EBF was based on point-in-time assessment (24 h recall). This might have possibly introduced some bias into the data, since children might have been fed with non-recommended food in previous times but not within the 24 h time frame.

## Conclusions

Our study offers new findings in the context of Nigeria, showing that maternal IPV experiences, particularly, psychological and physical abuse around the time of pregnancy and postpartum period, have a negative association with the likelihood of EBF for children under the age of 6 months. The policy implications arising in the light of this border on encouraging a system that does not stigmatize the victims of sexual abuse, so that the “culture of silence” does not force them to suffer in silence. Additionally, the providers of maternal healthcare services, specifically antenatal and postnatal care, should be adequately trained to discern and screen for the case of IPV, as well as how and where to refer cases for appropriate help. Furthermore, the patients must also be in a state of readiness to get the necessary help, and have agency over their own lives. While longitudinal studies may still be needed to help offer better insights on this relationship, future surveys should also endeavor to dissociate abuse experienced during pregnancy and postpartum so that future studies could look into differentiating the magnitude of association for both.

## Data Availability

The dataset used for analysis and reaching the conclusions of this study is available online at MEASURE DHS (https://www.dhsprogram.com/data/availabledatasets.cfm). They are released upon request subject to approval.

## Abbreviations

CI: Confidence Interval
CTS: Conflict Tactics Scale
EBF: Exclusive Breastfeeding
IPV: Intimate Partner Violence
LMICs: Low and Middle-Income Countries
MAR: Missing at Random
MCMC: Markov Chain Monte Carlo
DHS: Demographic and Health Survey
NPC: National Population Commission
PSU: Primary Sampling Units
SD: Standard Deviation
VIF: Variance Inflation Factors
WHO: World Health Organization

## References

[1] Takahashi K, Ganchimeg T, Ota E, Vogel JP, Souza JP, Laopaiboon M (2017). Prevalence of early initiation of breastfeeding and determinants of delayed initiation of breastfeeding: secondary analysis of the WHO global survey. Sci Rep.

[2] World Health Organization (2017). 10 facts on breastfeeding.

[3] World Health Organization (2001). Global strategy for infant and young child feeding, the optimal duration of exclusive breastfeeding.

[4] World Health Organization (2002). Infant and young child nutrition: Global strategy on infant and young child feeding.

[5] World Health Organization (2011). Media centre.

[6] Khan MN, Islam MM (2017). Effect of exclusive breastfeeding on selected adverse health and nutritional outcomes: a nationally representative study. BMC Public Health.

[7] Karamagi CAS, Tumwine JK, Tylleskar T, Heggenhougen K (2007). Intimate partner violence and infant morbidity: evidence of an association from a population-based study in eastern Uganda in 2003. BMC Pediatr.

[8] Hahn-Holbrook J, Schetter CD, Haselton M, Spiers M, Geller P, Kloss J (2013). Breastfeeding and maternal mental and physical health. Women’s Health Psychology.

[9] Campbell JC (2002). Health consequences of intimate partner violence. Lancet..

[10] Cerulli C, Talbot NL, Tang W, Chaudron LH (2011). Co-occurring intimate partner violence and mental health diagnoses in perinatal women. J Women's Health.

[11] Al-Modallal H (2016). Effect of intimate partner violence on health of women of Palestinian origin. Int Nurs Rev.

[12] Richardson R, Nandi A, Jaswal S, Harper S (2020). The effect of intimate partner violence on women’s mental distress: a prospective cohort study of 3010 rural Indian women. Soc Psychiatry Psychiatr Epidemiol.

[13] Ali PA, Dhingra K, McGarry J (2016). A literature review of intimate partner violence and its classifications. Aggress Violent Behav.

[14] National Population Commission (NPC) [Nigeria] and ICF International (2014). Nigeria Demographic and Health Survey 2013.

[15] Yount KM, DiGirolamo AM, Ramakrishnan U (2011). Impacts of domestic violence on child growth and nutrition: a conceptual review of the pathways of influence. Soc Sci Med.

[16] Mueller I, Tronick E (2019). Early life exposure to violence: developmental consequences on brain and behavior. Front Behav Neurosci.

[17] Scheeringa MS, Zeanah CH (1995). Symptom expression and trauma variables in children under 48 months of age. Infant Ment Health J.

[18] Bogat GA, DeJonghe E, Levendosky AA, Davidson WS, Von Eye A (2006). Trauma symptoms among infants exposed to intimate partner violence. Child Abus Negl.

[19] Martin-de-las-Heras S, Velasco C, Luna-del-Castillo JD, Khan KS (2019). Breastfeeding avoidance following psychological intimate partner violence during pregnancy: a cohort study and multivariate analysis. BJOG An Int J Obstet Gynaecol.

[20] Silverman JG, Decker MR, Reed E, Raj A (2006). Intimate partner violence around the time of pregnancy: association with breastfeeding behavior. J Women's Health.

[21] Sipsma HL, Magriples U, Divney A, Gordon D, Gabzdyl E, Kershaw T (2013). Breastfeeding behavior among adolescents: initiation, duration, and exclusivity. J Adolesc Health.

[22] James JP, Taft A, Amir LH, Agius P (2014). Does intimate partner violence impact on women’s initiation and duration of breastfeeding?. Breastfeed Rev.

[23] Finnbogadóttir H, Thies-Lagergren L (2017). Breastfeeding in the context of domestic violence a cross-sectional study. J Adv Nurs.

[24] Metheny N, Stephenson R (2020). Is intimate partner violence a barrier to breastfeeding? An analysis of the 2015 Indian National Family Health Survey. J Fam Violence.

[25] Islam MJ, Baird K, Mazerolle P, Broidy L (2017). Exploring the influence of psychosocial factors on exclusive breastfeeding in Bangladesh. Arch Womens Ment Health.

[26] Misch ES, Yount KM (2014). Intimate partner violence and breastfeeding in Africa. Matern Child Health J.

[27] UNESCO. Education Literacy rate(2018)*.* http://data.uis.unesco.org/. Accessed 20 Aug 2019.

[28] Kirk-Greene M, Hamilton A, Ade Ajayi JF, Udo RK, Falola TO (2019). Nigeria**.** Encycl*. Br*.

[29] Strauss MA (1979). Measuring intrafamily conflict and violence: the conflict tactics (CT) scales. J Marriage Fam.

[30] Kishor S, Johnson K (2004). Profiling domestic violence: a multi-country study.

[31] Benebo FO, Schumann B, Vaezghasemi M (2018). Intimate partner violence against women in Nigeria: a multilevel study investigating the effect of women’s status and community norms. BMC Womens Health.

[32] Rahman M, Nakamura K, Seino K, Kizuki M (2012). Intimate partner violence and use of reproductive health services among married women: evidence from a national Bangladeshi sample. BMC Public Health.

[33] Sunmola AM, Sambo MN, Mayungbo OA, Morakinyo LA. Moderating effect of husband’s controlling attitudes on the relation between women’s household decision-making autonomy and intimate partner violence experience in Nigeria. J Interpers Violence. 2019. 10.1177/0886260519888534. 10.1177/0886260519888534 31789086

[34] Weitzman A (2018). Does increasing women’s education reduce their risk of intimate partner violence? Evidence from an education policy reform. Criminology..

[35] World Health Organization (2001). Putting women first: ethical and safety recommendations for research on domestic violence against women.

[36] Tan KL (2011). Factors associated with exclusive breastfeeding among infants under six months of age in peninsular Malaysia. Int Breastfeed J.

[37] Mensah KA, Acheampong E, Anokye FO, Okyere P, Appiah-Brempong E, Adjei RO (2017). Factors influencing the practice of exclusive breastfeeding among nursing mothers in a peri-urban district of Ghana. BMC Res Notes.

[38] Ogbo FA, Eastwood J, Page A, Arora A, McKenzie A, Jalaludin B (2017). Prevalence and determinants of cessation of exclusive breastfeeding in the early postnatal period in Sydney. Int Breastfeed J.

[39] UCLA: Statistical Consulting Group. Multiple imputation in Stata https://stats.idre.ucla.edu/stata/seminars/mi_in_stata_pt1_new/ Accessed 10 Oct 2019.

[40] Koehler E, Brown E, Haneuse SJPA (2009). On the assessment of Monte Carlo error in simulation-based statistical analyses. Am Stat.

[41] Social Science Computing Cooperative. Multiple imputation in Stata: Estimating. (2012). https://www.ssc.wisc.edu/sscc/pubs/stata_mi_estimate.htm. Accessed 12 Oct 2019.

[42] Kendall-Tackett KA (2007). Violence against women and the perinatal period: the impact of lifetime violence and abuse on pregnancy, postpartum, and breastfeeding. Trauma Violence Abuse.

[43] Klingelhafer SK (2007). Sexual abuse and breastfeeding. J Hum Lact.

[44] Zureick-Brown S, Lavilla K, Yount KM (2015). Intimate partner violence and infant feeding practices in India: a cross-sectional study. Matern Child Nutr.

[45] Heise L, Ellsberg M, Gottmoeller M (2002). A global overview of gender-based violence. Int J Gynecol Obstet.

[46] D’Apolito K (2013). Breastfeeding and substance abuse. Clin Obstet Gynecol.

[47] Urke HB, Mittelmark MB (2015). Associations between intimate partner violence, childcare practices and infant health: findings from demographic and health surveys in Bolivia**,** Colombia and Peru. BMC Public Health.

[48] Antai D (2011). Controlling behavior, power relations within intimate relationships and intimate partner physical and sexual violence against women in Nigeria. BMC Public Health.

[49] Adams D (1990). Identifying the assaultive husband in court: you be the judge. Response to the Victimization of Women & Children..

[50] Kong SKF, Lee DTF (2004). Factors influencing decision to breastfeed. J Adv Nurs.

[51] Caleyachetty R, Uthman OA, Bekele HN, Martín-Cañavate R, Marais D, Coles J, et al. Maternal exposure to intimate partner violence and breastfeeding practices in 51 low-income and middle-income countries: a population-based cross-sectional study. PLoS Med. 2019;16. 10.1371/journal.pmed.1002921 PMC6771984 31574100

[52] Abrahams N, Jewkes R, Hoffman M, Laubsher R (2004). Sexual violence against intimate partners in Cape Town: prevalence and risk factors reported by men. Bull World Health Organ.

[53] Ajuwon AJ, Adegbite O (2008). Ethical and methodological challenges involved in research on sexual violence in Nigeria. Res Ethics.

[54] Davis RC, Taylor BG (1997). A proactive response to family violence: the results of a randomized experiment. Criminology..

[55] Kidman R (2017). Child marriage and intimate partner violence: a comparative study of 34 countries. Int J Epidemiol.

[56] Peterman A, Bleck J, Palermo T (2015). Age and intimate partner violence: an analysis of global trends among women experiencing victimization in 30 developing countries. J Adolesc Health.

